# Prevalence and behavior regarding cigarette and water pipe smoking among Syrian undergraduates

**DOI:** 10.1016/j.heliyon.2020.e05423

**Published:** 2020-11-05

**Authors:** Homam Alolabi, Mhd Obai Alchallah, Fatema Mohsen, Mosa Shibani, Hlma Ismail, Mhd Amin Alzabibi, Bisher Sawaf

**Affiliations:** a Faculty of Medicine, Syrian Private University, Damascus, Syria; b Faculty of Medicine, American University of Beirut Medical Center Beirut, Lebanon; c Internal Medicine Department, Hamad General Hospital, Hamad Medical Corporation, Doha, Qatar

**Keywords:** Education, Epidemiology, Public health, Smoking, Substance abuse and dependence, Attitude, Water pipe, Cigarette, Fagerström test, University students, War, Syria

## Abstract

**Background:**

The devastating Syrian crisis has raised concern regarding the social acceptance of smoking especially with water pipe use becoming a growing epidemic. We aim to determine the prevalence of cigarette and water pipe smoking among university students, along with identifying the addictive behavior among university students.

**Methods:**

A cross-sectional study was conducted at the Syrian Private University during World No-Tobacco Day, in Damascus, Syria during the war crisis. The survey consists of 4 sections: socio-demographic information, Fagerström Test of Nicotine Dependence, attitude towards water pipes, and perspective about smoking. Data were analyzed using the Statistical Package for Sciences version 25.0 (SPSS Inc., United States.)

**Results:**

Of the 622 participants, 429 (69%) were males with a mean age of 21.3 ± 3.1 years. The prevalence of tobacco smoking was 320 (51.4%), 208 (23.8%) for cigarettes, and 112 (18.0%) for water pipe. Smoking was significantly higher among male non-medical university students. The majority were low to moderate dependent when assessed by the Fagerström Test of Nicotine Dependence.

**Conclusions:**

This study showed a very high prevalence of smoking indicating the need for smoking cessation programs, access to effective quitting treatments, and mass media campaigns to diminish smoking among the youth.

## 1 Introduction

An estimated 80% of smokers live in low and middle-income countries [1]. The Middle East (ME) has a high prevalence of smoking, Jordan (35.0%), Saudi Arabia (30.4%), and Lebanon (26.3%), respectively [2]. In 2007 a study conducted at the same institution as the current study showed that 20.7% of students smoke [3], during the crisis (2014) another study in a governmental university reported 24.7% smoke cigarettes, and 30.4% smoke water pipe [4].

There were 14731 tons of tobacco manufactured in Syria in 2014, mostly for local consumption and partially for exportation. Alhamraa is the main cigarette product produced and sold in Syria, however there are other less popular brands including Ebla and Sharq. The price of a packet of cigarettes is 800–900 Syrian pounds (SYP), which is equivalent to almost a third of a dollar. Tobacco products are mainly sold in: street vendors (kiosk), convenience stores, grocery stores (supermarket), liquor stores, and tobacco stores [4, 5].

Tobacco comes in many forms, however; water pipes (also called hookah, narghila, argileh, hubble bubble, goza, and shisha, e.g. a clay bowl filled with tobacco, where smoke is inhaled through a tube) traditionally originated from ME, have been used for centuries, are now marketed worldwide [6]. ME tobacco usage is on a steep rise especially with the introduction of fruit-flavorings (ma'assel) in the early 1990s, and social acceptance increasing the appeal of water pipe smoking to younger generations [2], where cigarettes are becoming less fashionable nowadays [6]. Even though water pipe smoking has 10 times the amount of nicotine compared with cigarettes, and equivalence of smoking 50–100 cigarettes in a single session, it still contains many of the same toxicants (e.g. nicotine, and carbon monoxide) as cigarette smoking [7, 8]. Water pipe smoking is considered by Syrians as a pleasurable, and sociable experience among all ages of the society, while cigarette smoking is seen as a personal addiction [9]. Nowadays water pipes are available in most cafes, restaurants, hotels, and households, with catastrophically low prices and many restaurants offering it free of charge when purchasing a drink or a meal [5]. Integrated social and environmental frameworks of public health suggest that many factors, such as parental education, household income, illicit drug abuse, and alcohol consumption may be associated with water pipe tobacco use. As a conservative country illicit drug use and alcohol consumption is shunned upon in an Islamic country where approximately 90% of the population is Muslim [10]. Nevertheless, drug abuse has always been popular among prisoners, and with the brutal armed conflict leaving many regions out of control, increasing the smuggling of many illicit drugs [11]. Assessing these harmful factors is valuable to target high-risk groups through community-based interventions such as education, cessation campaigns, and social media [7]. Since many first initiated smoking during adolescence [12], it's a precious opportunity to study students' behavior regarding tobacco use, in order to prevent premature morbidity and mortality related to smoking [13], as students should be the main target of tobacco prevention programs in universities.

During this violent war, data on explosive weapon use and contamination around Damascus and Rural Damascus have reported a minimum of 94,792 munitions use based on 16,147 conflict events between 2013 and 2019. Various types of munitions include rockets, artillery shells, mortar shells, airplane launched munitions, helicopter-dropped barrel bombs, landmines, and suicide bombing vehicles. Destruction of oil refineries, power stations, pharmaceuticals, and plastic factories released hazardous toxins through the air. Civilians have therefore been exposed to explosive remnants of toxic smoke mixtures of silica, asbestos, heavy metals, fuels, debris, and rubble, thrown up by blast and linger over the cityscape like a shroud. This war toxication along with tobacco smoking hinders the community's development long after the cease of war.

This is the first study to demonstrate the addictive behavior of tobacco usage among Syrian students, which are still enduring suffering pain, and economic collapse. We aim to determine the prevalence of cigarette and water pipe smoking among SPU students and assess the nicotine dependence on cigarette smokers, and identify the habits, attitudes, and practices related to smoking behaviors. The objective of this study was to explore the patterns of smoking across schools, gender, and dual users (cigarette and water pipe smokers) and their association with the Fagerström test, and their attitudes and habits.

## 2 Methods

### 2.1 Study design, setting, and participants

This cross-sectional study was conducted using a convenience sampling method during World No-Tobacco Day (31/05/2019), at Syrian Private University (SPU) in Damascus, Syria. A written consent was obtained from all participants. Students were informed their participation was voluntary, all of the responses were recorded anonymously, response to all questions was not mandatory, and were allowed to opt-out of the survey at any time. Ethical approval was obtained from the Institutional Review Board (IRB), Faculty of Medicine, Syrian Private University. A structured self-completed English-language questionnaire was distributed among participants. The questionnaire contained 4 sections (22 questions): socio-demographic information (5 questions) including age, gender, current residence, and educational year; Fagerström Test of Nicotine Dependence is a widely used and validated six-item questionnaire (Heatherton, Kozlowski, Frecker, & Fagerström, 1991) [14], a score of 4 or more (nicotine dependence), and 6 or more (severe nicotine dependence) was adopted; attitude towards water pipes (4 questions); smoking attitude and habits (4 questions). The questionnaire is available as a supplementary file.

### 2.2 Statistical analysis

Data were analyzed using the Statistical Package for Social Sciences version 25.0 (SPSS Inc., Chicago, IL, United States) and reported as frequencies and percentages (for categorical variables) or means, medians, and standard deviations (SD) (for continuous variables). Chi-square test and one-way ANOVA test were applied to assess 2 objectives: 1. The relationships between dual smokers and both cigarette, and water pipe smokers regarding the Fagerström test, and their attitudes and habits 2. The gender differences regarding cigarette and water pipe smoking, nicotine dependence, and number of cigarettes smoked per day. *p-values* < 0.05 were considered statistically significant.

## 3 Results

### 3.1 Socio-demographic characteristics

Of 622 undergraduate students who filled the questionnaire, 429 (69%) were males, and 193 (31%) were females, Participants' ages ranged from 18 and 30 years, with a mean of 21.3 ± 3.1 years. The majority were 22 years or below 469 (75.4%), and living with their families 460 (74%); however, 66 (10.6%), and 77 (12.4%) were living with their friends, or alone respectively. A total of 320 (51.4%) students smoke: 143 (23%) only cigarettes, 112 (18.0%) only water pipes, and 65 (10.4%) both (Table 1).

**Table 1:** Socio-demographic characteristics: (n = 622).

**Age (years)**	22 and under	469 (75.4%)	**Accommodation**	Family	460 (74%)
	>22	153 (24.6%)		Friends	66 (10.6%)
**Gender**	Male	429 (69%)		Alone	77 (12.4%)
	Female	193 (31%)		University accommodation	1 (0.2%)
**Faculty**	Non-medical	155 (24.9%)		Other	18 (2.9%)
			**Smokers**	Both	65 (10.4%)
	Medical	467 (75.1%)		Only cigarette	143 (23%)
				Only water pipe	112 (18%)

### 3.2 Cigarette section

**Figure 1:**
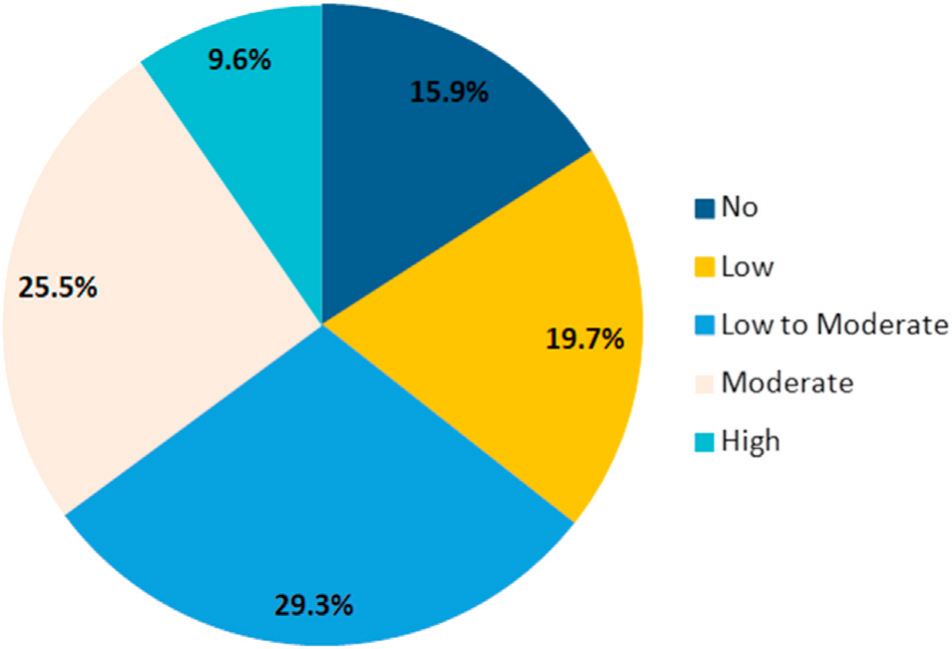
Pie Chart illustrate the Results of Fagerström Test of Nicotine Dependence.

We used the Fagerström Test of Nicotine Dependence in our study. The results revealed that the majority 61 (29.3%) of smokers were low to moderate dependence, 20 (9.7%) were high dependence, and 33 (15.9%) of students were not dependent, with a mean score of 3.6 (Figure 1). 61 (29.3%) smoke their first cigarette after an hour from waking up, 81 (38.9%) smoke 11–20 cigarettes per day, and 31 (14.9%) smoke more than 31 cigarettes per day.

Whereas 67 (32.2%) find it hard to stop smoking in prohibited sites, only 67 (32.2%) find it hard to quit their morning cigarette (Table 2).

**Table 2:** Cigarette smoking (Fagerström test) [17] questions: (n = 208).

		Cigarettes only (n = 143)	Dual users (n = 65)			Cigarettes only (n = 143)	Dual users (n = 65)
**How much time do you take to smoke after you wake up**	During 5 min	28 (19.6%)	10 (15.4%)	**How many cigarettes do you smoke a day?**	Less than 10	26 (18.2%)	25 (38.5%)
	6–30 min	49 (34.2%)	16 (24.6%)		11–20	61 (42.6%)	20 (30.7%)
	31–60 min	33 (23.1%)	11 (16.9%)		21–30	35 (24.5%)	10 (15.4%)
	>60 min	33 (23.1%)	28 (43.1%)		>31	21 (14.7%)	10 (15.4%)
**Do you find it hard to stop smoking in prohibited smoking areas?**	Yes	47 (32.9%)	20 (30.8%)	**Do you smoke more in the first hours of the morning?**	yes	46 (32.2%)	18 (27.7%)
	No	96 (67.1%)	45 (69.2%)		No	97 (67.8%)	47 (72.3%)
**What is the hardest cigarette to quit?**	The first cigarette in the morning	47 (32.9%)	20 (30.8%)	**Do you smoke if you are sick?**	Yes	55 (38.5%)	23 (35.4%)
	Other	96 (67.1%)	45 (69.2%)		No	88 (61.5%)	42 (64.6%)

There was no significant relationship found when assessing dependence to nicotine with dual users and single users, their type of residence (Table 1), and frequency of water pipe use (Table3).

**Table 3:** Water Pipe usage attitude: (n = 177).

		Waterpipe only users (n = 112)	Dual users (n = 65)			Waterpipe only users (n = 112)	Dual users (n = 65)
**Do you possess a water pipe?**	Yes	89 (79.5%)	52 (80%)	**In comparison tocigarette smoking, water pipe smoking is:**	Better	48 (42.9%)	28 (43%)
	No	23 (20.5%)	13 (20%)		Worse	29 (25.9%)	17 (26.3%)
**How often do you smoke a water pipe?**	Once a week	45 (40.2%)	26 (40%)		The same	35 (31.2%)	20 (30.7%)
	Twice or more a week	30 (26.8%)	17 (26.1%)	**Do you think smoking a water pipe is a good solution to quit cigarette smoking?**	Yes	26 (23.2%)	15 (23.1%)
	Once a day	22 (19.6%)	13 (20%)		No	52 (46.3%)	30 (46.2%)
	More than once a day	15 (13.4%)	9 (13.9%)		Don't know	34 (30.5%)	20 (30.8%)

### 3.3 Attitude towards water pipe smoking

Data showed that 141 (79.7%) of water pipe smokers possess their own water pipe, the majority 71 (40.1%) water pipe smoke once a week, 47 (26.6%) twice or more a week, and 35 (19.7%) more than once daily. When asked about their opinion on water pipes, 76 (42.9%) said it is better than cigarettes, and only 46 (25.9%) said that it is worse. 82 (46.3%) disagreed with water pipes being a good solution to quit cigarette smoking (Table 3).

There was no significant relationship found when assessing attitudes regarding water pipe smoking with dual users and water pipe smokers (single users).

### 3.4 Smoking attitude and habits

Students were asked about their self-opinion as smokers, the majority of students 191 (59.7%) stated that they know smoking is dangerous but they still enjoy it, whereas 91 (28.4%) said that they are not satisfied with smoking but find it difficult to quit, and 38 (11.9%) considered smoking as a good practice. When asked about their expenditure, 136 (42.5%) spend less than 10,000 SYP/month (~$23) and only 24 (7.5%) spend more than 45,000 SYP/month (~$104).

131 (41.2%) stated that they started smoking before the age of 17. We permitted students to select multiple answers in the following questions, where data revealed that 170 (53.1%) students have parents who smoke, and 124 (38.8%) students have siblings who smoke (Table 4). The most common reasons chosen by students for smoking were: habit and addiction 156 (48.7%), and pressure relief 118 (36.8%) (Figure 3) (Figure 4). There was no statistical significance among these three groups and their attitudes and habits regarding smoking.

**Table 4:** Smoking attitude and habits (n = 320).

		Cigarettes only (n = 143)	Waterpipe only (=112)	Dual users (n = 65)			Cigarettes only (n = 143)	Waterpipe only (=112)	Dual users (n = 65)
**What is your self-opinion as a smoker?**	I don't consider smoking as a wrong practice	11 (7.7%)	18 (16.1%)	9 (13.8%)	**Age of first smoke (years)**	<17	65 (45.5%)	39 (34.8%)	27 (41.5%)
	Not satisfied, but I find it difficult to quit smoking smoking	55 (38.5%)	18 (16.1%)	18 (27.7%)		18–19	58 (40.6%)	52 (46.4%)	29 (44.6%)
	I know the health dangers, but I enjoy smoking	77 (53.8%)	76 (67.8%)	38 (58.5%)		20–22	17 (11.8%)	16 (14.3%)	8 (12.3%)
**How much do you spend monthly in Syrian pounds on smoking products?**	<10 thousand	36 (25.2%)	77 (68.7%)	23 (35.4%)		>22	3 (2.1%)	5 (4.5%)	1 (1.5%)
	10-15 thousand	49 (34.5%)	24 (21.4%)	19 (29.2%)	**Do you have a positive family history of smokers? (more than one answer)**	One or both parents	70 (48.9%)	66 (58.9%)	34 (52.3%)
	15-45 thousand	43 (30.1%)	8 (7.2%)	17 (26.2%)		One or more siblings	49 (34.2%)	51 (45.5%)	24 (36.9%)
	>45 thousand	15 (10.5%)	3 (2.7%)	6 (9.2%)		None	43 (30.1%)	18 (16.1%)	19 (29.2%)

### 3.5 Gender differences

**Figure 2:**
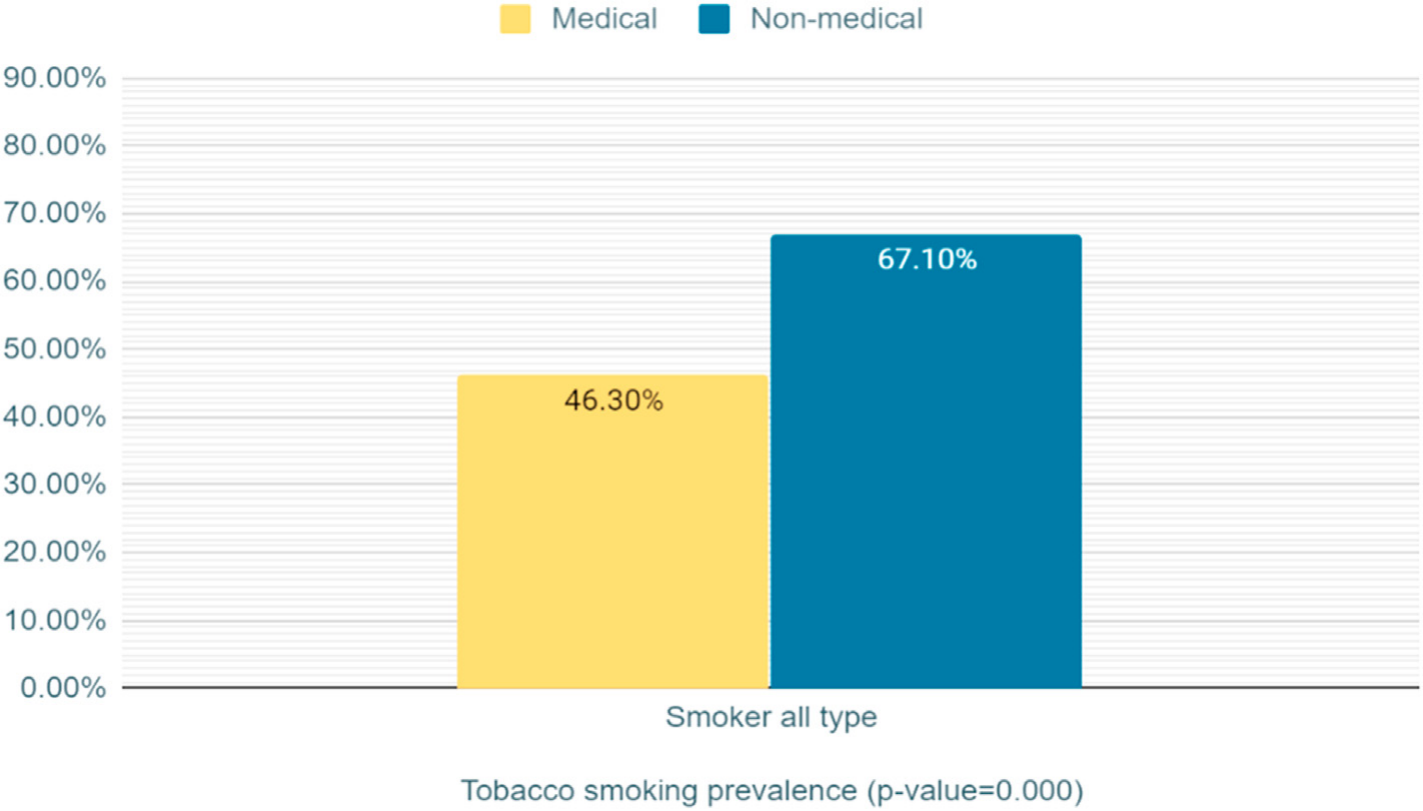
Bar Graph illustrate the tobacco use prevelance among medical and non-medical university students.

**Figure 3:**
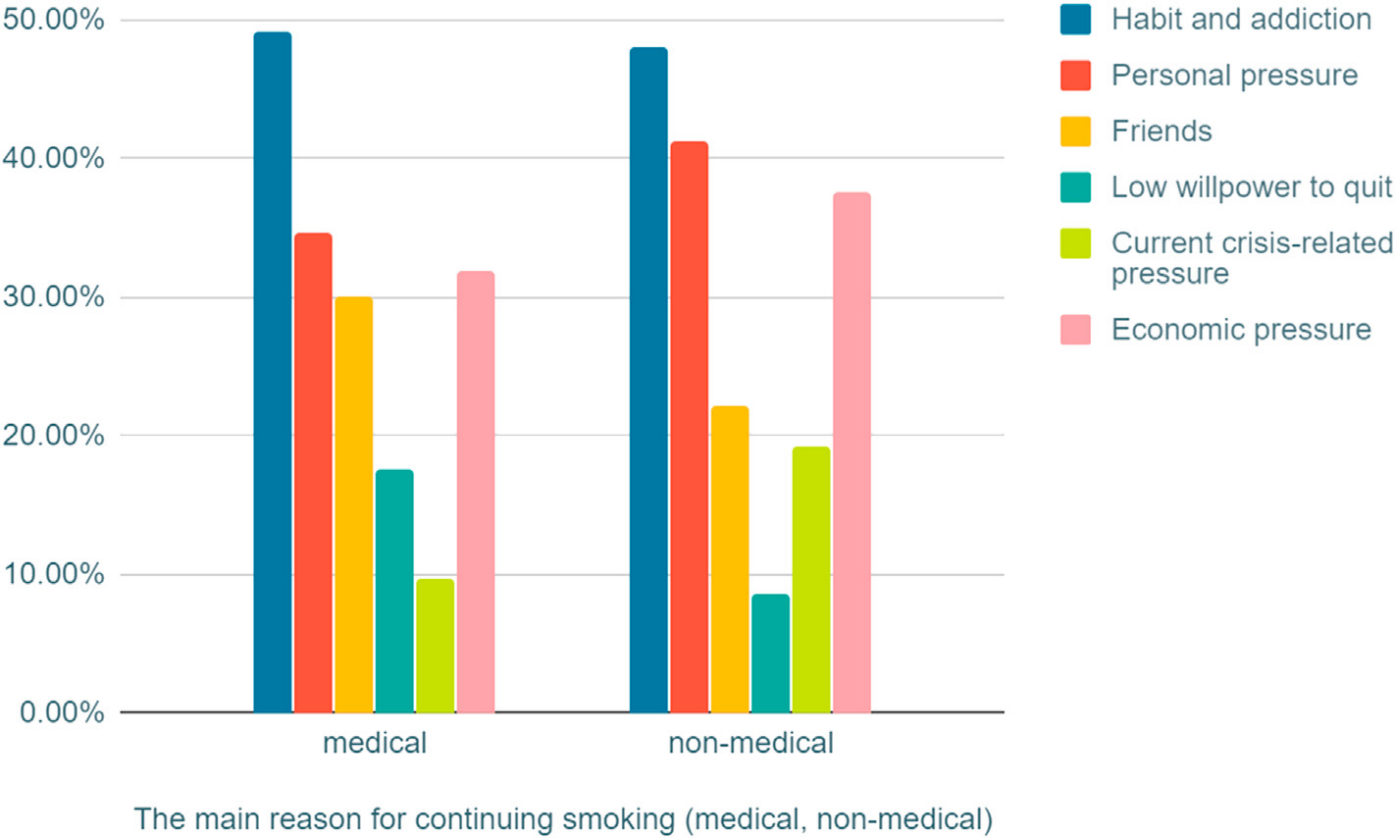
Comparing reason(s) for smoking between medical and non-medical students.

**Figure 4:**
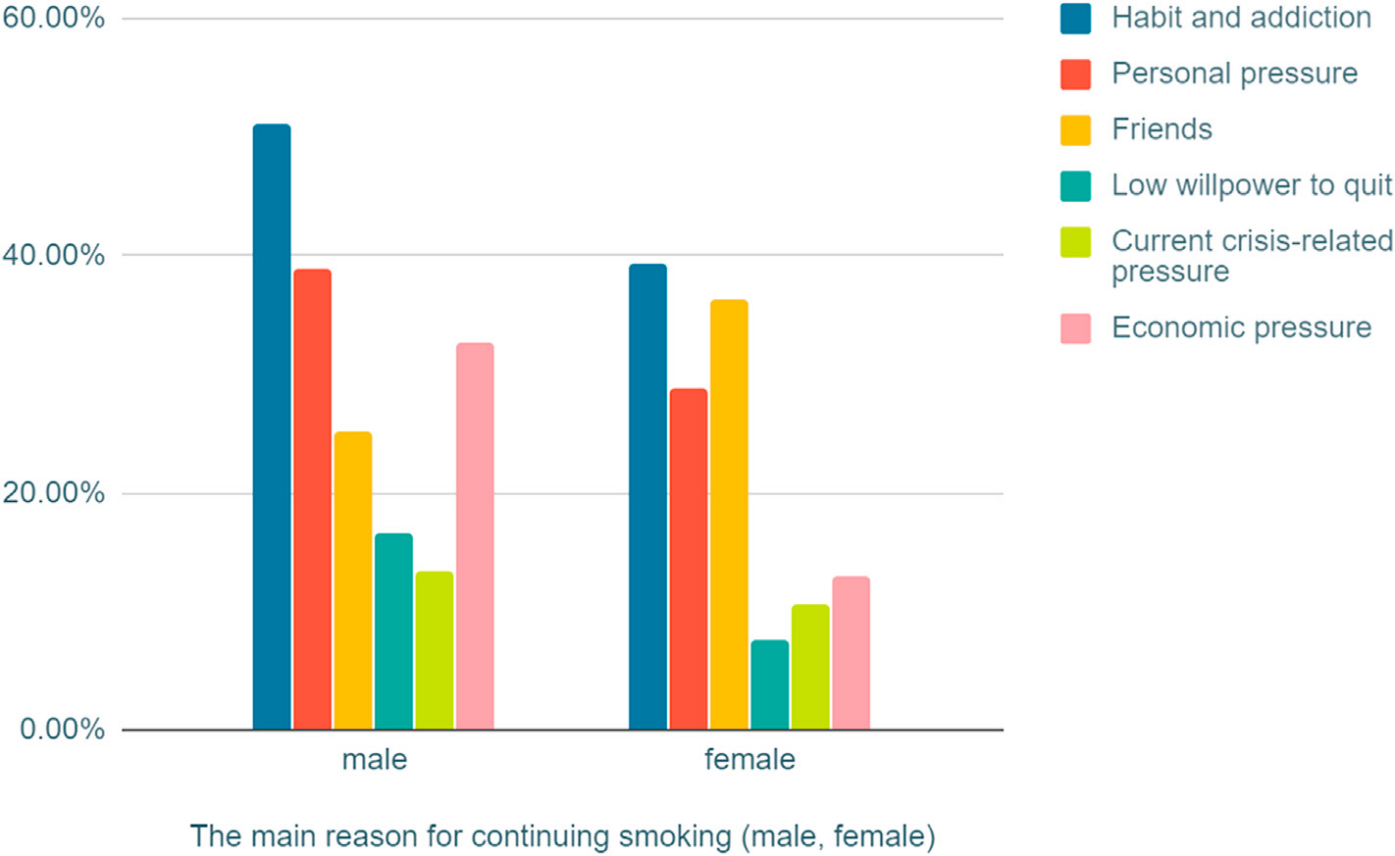
Reason(s) for smoking between male and female students.

Data revealed significantly more males smoke cigarettes compared with females (p < 0.0001), while significantly more females smoke water pipe compared with males (p < 0.0001) (Table 5). The prevalence of smoking in medical students is significantly lower than non-medical students (p < 0.0001) (Figure 2). Habit and addiction were found to be the most common reason for smoking (Figure 3) (Figure 4).

**Table 5:** Relationship between smoking prevalence, dependency, and gender (Bold font indicate statistical significance).

		Male (n = 429)	Female (n = 193)	Total (n = 622)
**Cigarette**	Smokers	**193 (44.9%)**	15 (7.7%)	208 (33.4%)
	Non-smokers	236 (55.1%)	178 (92.2%)	414 (66.5%)
**Water pipe**	Smokers	96 (22.4%)	**81 (41.7%)**	177 (28.4%)
	Non-smokers	333 (77.6%)	112 (58%)	445 (82%)

		**Male cigarette smokers** (n = 193)	**Female cigarette smokers** (n = 15)	**Total cigarette smokers** (n = 208)
**Nicotine dependency Score**	No dependence	24 (12.4%)	9 (60%)	33 (15.9%)
	Low dependence	38 (19.7%)	3 (20%)	41 (19.7%)
	Low to mod dependence	**60 (31.1%)**	1 (6.7%)	61 (29.3%)
	Moderate dependence	51 (26.4%)	2 (13.3%)	53 (25.5%)
	High dependence	20 (10.4%)	0 (0%)	20 (9.6%)
**Number of daily smoked cigarettes**	≤10	39 (20.2%)	12 (80%)	51 (8.2%)
	11–20	**78 (40.4%)**	3 (20%)	81 (13%)
	21–30	45 (23.3%)	0	45 (7.2%)
	≥31	31 (16.1%)	0	31 (5%)

Males who smoked 11–20 cigarettes per day were significantly higher compared with those who smoked ≤10, 21–30, and ≥31 (p < 0.0001). (Table 5).

## 4 Discussion

Tobacco use is a major concern worldwide, with high morbidity and mortality, more efforts should be made to stop the growing epidemic [15]. The prevalence of smoking in our study is 320 (51.4%), of which 208 (33.4%) are cigarette smokers. Our findings are higher than a study conducted during the crisis [4], and much higher than studies reported pre-war [16, 17] Neighboring studies revealed lower prevalence of smokers compared with our study: Jordan, Iraq, Lebanon, and Turkey [18, 19, 20, 21].This high prevalence of smoking among Syrian undergraduates can be attributed to the psychological impacts of the catastrophic war. A study proved that depression has risen to 43.9% among Syrian refugees compared to 6.5% pre-war, leading to the adoption of smoking behaviors [22, 23].

The prevalence of tobacco smoking among non-medical students was significantly higher than medical students, this is in line with a previous study at the same institution [3]. Medical students are known to be educated about tobacco related diseases and the long term effects of tobacco use on the human body. As future doctors-the role models of our society-they play a pivotal role in increasing awareness of the hazardous effects of smoking and are frontline in aiding cessation campaigns. Also, the financial situation may vary between students of different faculties, as a private school, medical students tend to pay at least twice more than other faculties, this means that only families of well-income can afford to place their children in medical school.

We measured our students' dependence to nicotine by the Fagerström test, In our study, the majority of students were low to moderate dependent compared with a Turkish study that reported the majority as moderate to high nicotine dependent [21]. While the gender differences were insignificant in some studies [24, 25, 26, 27, 28], others reported higher dependence among males compared with females. The present study concurs that males are more addicted to nicotine. These findings are justified by the fact that nicotine intake differs between gender, women seldom consume as much nicotine as men, since it has been shown they are more sensitive to nicotine as well as they have a greater metabolism rate to nicotine during pregnancy and while taking Oral contraceptives [29, 30]. However, our study revealed that males smoke more cigarettes in comparison with females; Inconsistent with a previous study at the same institution, which stated that female students smoked significantly higher quantities of cigarettes per day compared with males [6]. The social stigma related to women smoking in the Arab countries is seen as masculine behavior, defeminizing women's traits; therefore, shameful and shunned upon in our society [31]. Half of the adolescents who experiment with smoking become regular smokers. Evidence has shown that the earlier the individual starts smoking the more likely they are to become regular adult smokers [32, 33]. The present study showed that the majority of undergraduates started smoking before the age of 19 These findings are in accordance to previous studies [34, 35, 36].

Water pipe smoking is extensively used in the ME and is now adopted by western countries [37, 38]. Since water pipe users inhale a large amount of nicotine and poisons [39], it's crucial to set limits and increase awareness about this atrocity. Our study astonishingly showed that 177 (28.5%) are current water pipe smokers, lower in comparison with studies conducted before and during the war [4], and in the ME [7, 18, 34, 40, 41]. Our study revealed a lower rate of water pipe smoking compared with cigarette smoking which can be attributed to the higher expenses of water pipe smoking; a water pipe session (1500 SYP) is 50% higher in cost than an average packet of cigarettes (1000 SYP). Our findings reveal that females smoke water pipe more than males. While other studies reported a male predominance in water pipe smoking [4, 42], a recent Syrian study among the whole population found a notable increase in female water pipe consumption compared with studies before the war [43]. Evidence showed that Arabic countries have less stigma associated with women's use of water pipes than with cigarettes [39].

Syria signed the Framework Convention on Tobacco Control (FCTC) in 2003; however, the implementation of the articles have been misconducted [5]. A low perception was found with regards to water pipe smoking in this study, parallel with no national quitline, free nicotine replacement therapy, or mass media cessation campaigns, concurs that drastic measures should be taken to implement the rules of the FCTC, and increase awareness amongst our community [4].

Syria is suffering a deep economic recession where estimated losses have reached $226 billion Gross Domestic Product between 2011 and 2016 [44]. Regarding expenditure, the majority of students spend less than 10,000 thousand SYP/month (~$23) and only a few spend more than 45,000 thousand SYP/month (~$104), these numbers seem low when shown in dollars but are in fact devastating to an average household income of a Syrian who earns 50,000 SYP/month (~$115). The financial distress this war has created is the perfect trigger for depression, which can initiate and exacerbate addiction to smoking [45].

## 5 Conclusion

The Syrian crisis has more than doubled the numbers of tobacco consumption (51.4%) amongst Syrian private university students. The majority revealed a low to moderate nicotine dependence. Water pipe smoking is highly prevalent among females, while males were associated with cigarette smoking. Water pipe smoking is increasingly becoming more popular among students and more socially acceptable as an alternative to cigarettes. Further research is required to identify the social, and environmental factors associated with smoking to target high-risk groups with cessation campaigns, and diminish smoking among the youth. Our students are not skillfully prepared to quit smoking, therefore introduction of smoke-free laws, improved access to effective quitting treatments, increase excise tax on tobacco products, and mass media campaigns are a necessity.

## 6 Limitation

The limitations recognized within this study include:

1. This cross-sectional study does not reflect the overall Syrian nation 2. The use of a questionnaire, which is a self-reported approach could lead to underreporting and recall bias. 3. Estimating sampling variability and identifying possible bias. To overcome these limitations, a further study on a national level is required. 4. This study primarily addressed a general description of the factors associated with smoking, further linear regression analysis to determine the factors associated/contributed to smoking is required.

## Declarations

### Author contribution statement

H. Alolabi and M. Alchallah: Analyzed and interpreted the data; Contributed reagents, materials, analysis tools or data; Wrote the paper.

F. Mohsen: Contributed reagents, materials, analysis tools or data; Wrote the paper.

M. Shibani, H. Ismail and M. Alzabibi: Conceived and designed the experiments; Performed the experiments; Contributed reagents, materials, analysis tools or data.

B. Sawaf: Conceived and designed the experiments; Contributed reagents, materials, analysis tools or data.

### Funding statement

This research did not receive any specific grant from funding agencies in the public, commercial, or not-for-profit sectors.

### Declaration of interests statement

The authors declare no conflict of interest.

### Additional information

No additional information is available for this paper.

## Appendix A Supplementary data

survey survey
