# Supplementary material for: Prevalence and behavior regarding cigarette and water pipe smoking among Syrian undergraduates
Source: Heliyon. 2020 Nov 5;6(11):e05423. doi: 10.1016/j.heliyon.2020.e05423 (PMC7653068; doi:10.1016/j.heliyon.2020.e05423)
Supplement: survey [file mmc1.docx]

Ethical approval of the questionnaire was obtained from the Institutional Review Board (IRB), Faculty of Medicine.

| **1.** Personal information: | | | |
| --- | --- | --- | --- |
| **Age (years)** | ⬜22 and under | **Accommodation** | ⬜Family |
|  | ⬜>22 |  | ⬜Friends |
| **Gender** | ⬜Male |  | ⬜Alone |
|  | ⬜Female |  | ⬜University accommodation |
| **Faculty** | ⬜Non-medical |  | ⬜ Other |
|  |  | **Do you smoke?** | ⬜Both |
|  | ⬜Medical |  | ⬜Only cigarette |
|  |  |  | ⬜Only water pipe |

| **2.** Cigarette smoking (Fagerström test)^1^ questions: | | | |
| --- | --- | --- | --- |
| **How much time do you take to smoke after you wake up** | ⬜During 5 min | **How many cigarettes do you smoke a day?** | ⬜Less than 10 |
|  | ⬜6-30 min |  | ⬜11-20 |
|  | ⬜31-60 min |  | ⬜21-30 |
|  | ⬜>60 min |  | ⬜>31 |
| **Do you find it hard to stop smoking in prohibited smoking areas?** | ⬜Yes | **Do you smoke more in the first hours of the morning?** | ⬜yes |
|  | ⬜No |  | ⬜No |
| **What is the hardest cigarette to quit?** | ⬜The first cigarette in the morning | **Do you smoke if you are sick?** | ⬜Yes |
|  | ⬜Other |  | ⬜No |

| **3.** Water Pipe questions: | | | |
| --- | --- | --- | --- |
| **Do you possess a water pipe?** | ⬜Yes | **In comparison to cigarette smoking, water pipe smoking is:** | ⬜Better |
|  | ⬜No |  | ⬜Worse |
| **How often do you smoke a water pipe?** | ⬜Once a week |  | ⬜The same |
|  | ⬜Twice or more a week | **Do you think smoking a water pipe is a good solution to quit cigarette smoking?** | ⬜Yes |
|  | ⬜Once a day |  | ⬜No |
|  | ⬜More than once a day |  | ⬜Don't know |

| **4.** Smoking attitude and habits questions : | | | |
| --- | --- | --- | --- |
| **What is your self-opinion as a smoker?** | ⬜I don't consider smoking as a wrong practice | **Age of first smoke (years)** | ⬜<17 |
|  | ⬜Not satisfied, but I find it difficult to quit smoking smoking |  | ⬜18-19 |
|  | ⬜I know the health dangers, but I enjoy smoking |  | ⬜20-22 |
| **How much do you spend monthly in Syrian pounds on smoking products?** | ⬜<10 thousand |  | ⬜>22 |
|  | ⬜10-15 thousand | **Do you have a positive family history of smokers?**  **(more than one answer)** | ⬜One or both parents |
|  | ⬜15-45 thousand |  | ⬜One or more siblings |
|  |  |  | ⬜None |
|  | ⬜>45 thousand |  |  |
|  |  |  |  |

1. Heatherton TF, Kozlowski LT, Frecker RC, Fagerström KO. The Fagerström Test for Nicotine Dependence: a revision of the Fagerström Tolerance Questionnaire. *British journal of addiction*. Sep 1991;86(9):1119-27. doi:10.1111/j.1360-0443.1991.tb01879.x
